# Nerve Biopsy in Peripheral Neuropathies: Not All Water Is under the Bridge

**DOI:** 10.3390/brainsci11050550

**Published:** 2021-04-27

**Authors:** Marco Luigetti, Andrea Di Paolantonio

**Affiliations:** 1 Fondazione Policlinico Universitario “A. Gemelli” IRCCS, UOC Neurologia, Largo A Gemelli 8, 00168 Rome, Italy; 2 Dipartimento di Neuroscienze, Università Cattolica del Sacro Cuore, 00168 Rome, Italy; andrea.dp1988@gmail.com

**Keywords:** peripheral neuropathies, nerve biopsy, nerve pathology, amyloid, CIDP, CMT

Sural nerve biopsy has long been a valuable diagnostic tool for the study of peripheral neuropathies, although the recent introduction of non-invasive techniques (e.g., neuroimaging techniques, skin biopsy) and advanced genetic and immunological testing has changed the diagnostic workup of peripheral nervous system diseases. Besides its diagnostic role, sural nerve biopsy has helped one understand the pathogenesis of several neuropathies, and, in selected cases with difficult clinical diagnosis, it continues to represent a useful and irreplaceable tool.

In chronic inflammatory demyelinating polyneuropathy, nerve biopsy shows a great pathological heterogeneity that may be strictly dependent on the disease phase or on the multifocality of inflammatory process, but it could also reflect different mechanisms of immune responses involved [1].

In hereditary transthyretin amyloidosis, sural nerve biopsy may occasionally show not only an axonal loss but also myelin alterations, suggesting a Schwann cell damage caused by toxic effect or by mechanical stress from the formation of amyloid fibrils [2].

In hematological diseases, sural nerve biopsy remains the gold standard for the diagnosis of vasculitis, neurolymphomatosis, and light chain amyloidosis [3].

Nerve biopsy is not only useful in the diagnosis of neuropathies: many investigations rely on nerve pathology to confirm their hypothesis about pathogenetic aspects of diseases [4] or even to devise new study protocols for animal models [5].

Eventually, nerve biopsy will not be the only technique available to study nerve morphology: there is an increasing interest in literature about ultrasounds, used not only as a diagnostic tool, but also to treat many mononeuropathies [6]; and about skin biopsy, easy to perform and somewhat better than nerve biopsy in studying small fibers [7].

The purpose of this Special Issue is to publish original research regarding pathological findings in different types of peripheral neuropathies which can contribute to help clinicians in final diagnosis.

## Data Availability

Not applicable.
